# Health-care services utilization and costs associated with radical cystectomy for bladder cancer: a descriptive population-based study in the province of Quebec, Canada

**DOI:** 10.1186/s12913-015-0972-3

**Published:** 2015-08-05

**Authors:** Fabiano Santos, Alice Dragomir, Ahmed Sayed Zakaria, Wassim Kassouf, Armen Aprikian

**Affiliations:** Division of Cancer Epidemiology, Department of Oncology, McGill University, 546 Pine Avenue West, Montreal, QC Canada; Division of Urology, Department of Urology, McGill University Health Centre, 1650 Cedar Avenue, Montreal, QC Canada

**Keywords:** Bladder cancer, Radical cystectomy, Health-services utilization, Costs

## Abstract

**Background:**

Bladder cancer (BC) has the highest lifetime treatment costs per patient of all cancers. The objective of this study was to characterize the use of health-care services and costs associated with BC among patients who underwent radical cystectomy (RC) in the province of Quebec.

**Methods:**

We conducted a descriptive study in a retrospective cohort of patients who underwent RC for BC between 2000 and 2009. Data was obtained from two health administrative databases (RAMQ and ISQ). We calculated average costs per patient and total costs in 2014 Canadian dollars for the following components of costs: 1) Pre-surgery costs (pre and post-urologist consultations, urologist consultations, cystoscopies, TURBTs, imaging procedures); 2) Costs of radical cystectomy and 3) Post-surgery costs (urologist consultations, post-operative consultations, medical oncologist consultations, imaging procedures and post-operative complication management). ARIMA models were used to evaluate trends in average costs per patient over the study period.

**Results:**

Among 2759 patients included in the study (75 % men), average pre-surgery costs, RC costs, and post-surgery costs were estimated at 3762$, 18979$ and 4770$, respectively. RC cost was responsible for 69 % of total costs, followed by post-operative consultations (7.8 %), post-operative complications and TURBTs (6 % of total costs, each). Academic hospitals performed RC at a lower average cost, compared to community hospitals (difference of $1000, p < .0001). A decreased trend in post-surgery costs was detected in the year 2009.

**Conclusions:**

Costs of RC, TURBT, consultations and post-operative complications were the most important economic components of total RC cost per patient in Quebec. Academic hospitals performed RC at a lower cost, compared to community hospitals.

## Background

Bladder cancer (BC) has the highest lifetime treatment costs per patient of all cancers, from diagnosis to death [1, 2]. Perioperative and post-operative complications, high recurrence rates, intensive surveillance strategies, and expensive treatment costs are the key contributors to the economic and health-services burden of this disease [3]. BC is the second most prevalent urological cancer worldwide and the fifth most common diagnosed malignancy in Canada, with 8000 incident cases and 2200 deaths expected for 2014 [4]. In the USA, 74 690 new cases and 15 580 deaths from BC are estimated for this year [5]. A quarter of patients present with muscle invasive BC, and about half of these individuals have occult distant metastases at the time of presentation [3]. Transurethral resection of the bladder tumor (TURBT) is a diagnostic and therapeutic intervention in cases of superficial disease, while muscle invasive bladder cancer often requires radical cystectomy (RC) [6]. Epidemiologic trends, aging and evolving population demographics along with technological advances in endoscopy, diagnostic and surgical technique will make the management of patients with bladder cancer in the near future a more substantial economic challenge [7, 8]. There is a lack of studies on the trajectories of BC patients in the continuum of care with respect to costs and the use of medical services [8]. Therefore, the objective of the present study was to describe health-care utilization and associated costs among patients who underwent RC for BC in the province of Quebec - Canada, as from the public healthcare system perspective.

## Methods

### Study design

We conducted a descriptive study analyzing health-care services utilization and costs associated with BC in a retrospective cohort of patients who underwent radical cystectomy in Quebec between the years 2000 and 2009.

### Data source

The cohort was built with the linkage of two provincial health administrative databases: the medical billing records database of the *Régie de l’assurance maladie du Québec* (RAMQ), and the *Fichier des évenements démographiques de l’Institut de la statistique du Québec* (ISQ). The RAMQ is the government body that administers healthcare provision in the province. All healthcare services are recorded in the RAMQ administrative databases and its associated claims files. The RAMQ claim file provides information on medical services dispensed to all Quebec residents (information on physician-based ICD-9 diagnostic codes, act codes for therapeutic procedures, their calendar dates and associated costs, characteristics of the patient, health care providers and hospital facilities). The ISQ administers the *Fichier des événements démographiques* which provides vital status data. The linkage between RAMQ and ISQ data is possible using a patients’ anonymous identifier (generated from the *Numéro d’assurance maladie* - NAM, which is a unique identifier for all legal residents of Quebec). The use of the data was approved by the *Comission de l’acces a l’information* (CAI) of Quebec. Ethics approval was obtained from the Research Ethics Board of the McGill University Health Centre.

### Study population

Patients were selected from January 1st 2000 to September 30th 2009. We excluded subjects aged less than 40 years-old (the age cut-off point for micro-hematuria workup in Canada) [9]. The index date is the date on which each patient entered into the cohort (calendar date of RC).

### Health-care services utilization

We identified health-care services utilization for patients who underwent RC for BC cancer during the 4 months period before RC. The 4 months’ time frame was adopted to assure that all medical services consumed by patients were associated to RC for BC. We identified the following components of health-care utilization: 1) Pre-surgery period (pre-and post-urologist consultations, urologist consultations, cystoscopies, TURBTs, pathology and imaging procedures), 2) Surgical period (RC), and 3) Post-surgery period (post-operative urologist, medical oncologist, and other consultations, and imaging procedures). Post-surgery health services were determined up to one year after surgery.

### Cost assignments

Analogously to the time frame adopted for the identification of health-care services, we calculated average costs per patient and total costs for the following components of costs on the continuum of care for BC : 1) Pre-surgery costs (cost of pre-urologist consultations, cost of post-urologist consultations, cost of urologist consultations, costs of cystoscopies, costs of TURBTs, costs of pathologic and imaging procedures; 2) Costs of RC, and 3) Post-surgery costs (costs of urologist consultations, costs of post-operative consultations, costs of medical oncologist consultations, costs of imaging procedures and costs of post-operative complications management). The unit cost of each medical service for consultations and imaging procedures was documented from the RAMQ’s list of medical procedures act codes approved for physician reimbursement fees in Quebec [10, 11]. We did not include costs associated to equipment and maintenance when computing costs for imaging procedures. When applicable, average and total costs for cystoscopy, TURBT and RC included estimation of hospitalization costs, pathology reports, urine cytology, anesthetist fees and surgical cardio-pulmonary monitoring. We assumed that each cystoscopy generates one urine cytology report; each TURBT leads to a same-day hospitalization stay and 1 pathology report, and each RC leads to 7 days hospitalization and 1 pathology report. Post-operative complications were identified by means of RAMQ procedure flash act codes, as described in previous work [12]. Hospitalization and pathology costs were estimated from the *Ministere de la Sante et des Services Sociaux (MSSS) du Quebec* and the McGill University Health Centre administration, respectively [13, 14]. The unit costs and sources are presented in Table 1. All costs were assigned in Canadian dollars and were estimated from the 2014 Quebec’s public healthcare system perspective.

**Table 1:** Costs associated with bladder cancer: Cost Components and Unit cost. Time horizon: from 4 months prior to radical cystectomy until 1 year after surgery^a^

Procedure	Unit cost^b^	Source
PRE-SURGERY		
Pre-urologist consultations		
• Physician fees	$ 50	Bladder cancer cohort
	Range: $ 15.20 – $ 94.4	
Post-urologist consultations		
• Physician fees	$ 50	Bladder cancer cohort
	Range: $ 15.20 – $ 94.4	
Urologist consultations		
• Physician fees	$ 40	Bladder cancer cohort
	Range: $16.9 – 45.6	
Cystoscopies		
• Physician fees	$ 50.9	RAMQ reimbursement act code list (10,11)
• Procedure fees	$ 192.3	Quebec MSSS (14)
• Urinary cytology	$ 87	MUHC administration
TURBT		
• Physician fees	$ 208	RAMQ reimbursement act code list (10,11)
• Hospitalization	$ 1371	Quebec MSSS (14)
• Anesthesia physician fees	$ 150	RAMQ reimbursement act code list (10,11)
• Pathology report	$ 40	MUHC administration
Imaging		
• Physician fees	$ 45	Bladder cancer cohort
	Range: $ 16.5 - $200	
RADICAL CYSTECTOMY		
• Physician fees	$ 1 880	RAMQ reimbursement act code list (10,11)
• Hospitalization	$ 14 855	Quebec MSSS (14)
• Anesthesia physician fees	$ 1 160	RAMQ reimbursement act code list (10,11)
• Pathology report	$ 450	MUHC administration
POST-SURGERY		
Post-operative consultations		
• Physician fees	$ 50	Bladder cancer cohort
	Range: $ 15.20 – $ 94.4	
Post-operative urologist consultations		
• Physician fees	$ 40	Bladder cancer cohort
	Range: $16.9 – 45.6	
Post-operative medical oncologist consultations		
• Physician fees	$ 39	Bladder cancer cohort
	Range: $ 16.9 - $ 98	
Post-operative imaging		
• Physician fees	$ 45	Bladder cancer cohort
	Range: $ 16.5 - $200	
Post-operative complications		
• Physician fees	$ 550	Bladder cancer cohort
	Range: $ 61.5 - $ 1184	
• Hospitalization	$ 1371	Quebec MSSS (14)

^a^Costs are shown in 2014 Canadian dollars

^b^Physician fees’ unit costs for consultations and imaging are expressed as average of unit cost according to each act code for reimbursement to different specialties and imaging procedures

### Covariates

We analysed BC-associated costs across two groups of variables: 1) Patient-related variables: age (four categories: less than 60, 60–69, 70–75, more than 75 years) and gender (dichotomous); and 2) Health-care services related variables: hospital facility where RC was performed, hospitals hosting an urology teaching program (dichotomous), surgeons’ annual RC case load (three categories: surgeons who perform less than 3 RC/year, surgeons who perform between 3–9 RC per year and surgeons who perform more than 9 RC/year), geo-administrative region where RC was performed (4 regions, grouped A to D) and calendar date of RC. Geo-administrative division is based on the MSSS’s Academic Integrated Network of Health which divides the province of Quebec into four regions according to provision of medical services and university affiliation [15].

### Statistical analyses

Demographic characteristics, age and gender-specific information of the study population was retrieved. Descriptive statistical analyses, including mean, median, standard deviation (SD) and range of costs were calculated, along with patient’s units of medical services related to each component of costs. Normality of data distribution was analysed by the Shapiro–Wilk test. Comparisons of average costs between groups were performed by t-tests and ANOVA tests, when applicable. We used autoregressive integrated moving average (ARIMA) models to evaluate trends in average total costs per patient, average radical cystectomy costs per patient and post-surgery costs per patient. We analysed differences between observed and forecasted average costs per patient over 117-month periods ranging from January 2000 to September 2009. Cut-off points for comparison between observed and forecasted values were established after seasonal trends inspection of the time series plot for each component of cost. Stationarity was assessed using the autocorrelation function and the augmented Dickey Fuller test. The autocorrelation, partial autocorrelation, and inverse autocorrelation graphic functions were used to model parameter appropriateness and seasonality. The presence of white noise was assessed by examining the autocorrelation at various lags using the Lung-Box chi squared test. All analyses were two-sided with p ≤ 0.05 being considered significant. SAS 9.3 (SAS Institute Inc., Cary, NC, USA) was used to conduct the calculations.

## Results

### Characteristics of the study cohort and the health-care system

Baseline characteristics of the cohort are summarized in Table 2. We analyzed a cohort formed by 2759 patients who underwent RC for BC and with medical services data available for the four months period before RC (75 % were men). Approximately 30 % of patients had post-operative complications. A total of 1355 patients (49 %) died during the study time period. Mean and median follow-up of the cohort was 34 months (standard deviation: 32 months) and 21.6 months (range: 1 day-118 months), respectively. The estimated overall 5-year survival rate was 46 %. During the study period, RCs were performed by 122 surgeons in 42 hospitals across the province. A total of 42 % of surgeries were performed in a hospital hosting a urology training program, whereas 43 % of procedures were performed in hospitals with an annual case load of <10 RCs per year. Surgeons with an annual RC case load of <3 RCs per year were responsible for 58 % of RCs.

**Table 2:** Characteristics of the study population

	Total (2759, 100 %)
Patient-related variables	
Gender	
Male	2091 (75.8 %)
Female	668 (24.2 %)
Age	
Less than 60 years-old	643 (23.3 %)
Between 60–69 years-old	828 (30 %)
Between 70–75 years-old	557 (20.2 %)
More than 75 years-old	731 (26.5 %)
Post-operative complications	
Yes	849 (30.7 %)
No	1910 (69.3 %)
Overall mortality	1355 (49.1 %)
5 years overall survival	46 %
Health system characteristics	
RUIS-MSSS region where surgery was performed	
Region A	1342 (48.6 %)
Region B	632 (23 %)
Region C	563 (20.4 %)
Region D	222 (8 %)
Surgery in a urology teaching hospital	1172 (42.48 %)
Hospital annual RC case load	
Less than 10 RCs per year	1184 (42.9 %)
Between 10–25 RCs per year	934 (33.8 %)
More than 25 RCs per year	641 (23.3 %)
Surgeon annual RC case load	
Less than 3 RCs per year	1621 (58.7 %)
Between 3–9 RCs per year	814 (29.6 %)
More than 9 RCs per year	324 (11.7 %)

### Health care services utilization and costs associated with bladder cancer: pre and post-RC operative period

**Fig 1:**
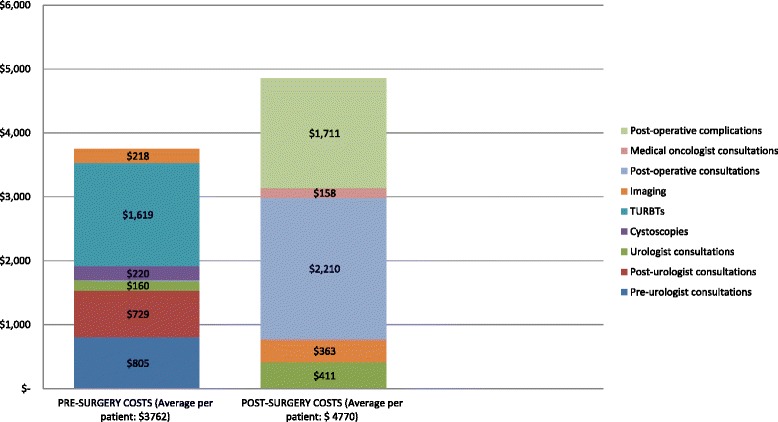
Weighted average costs per patient attributable to the health continuum of care for bladder cancer

**Fig 2:**
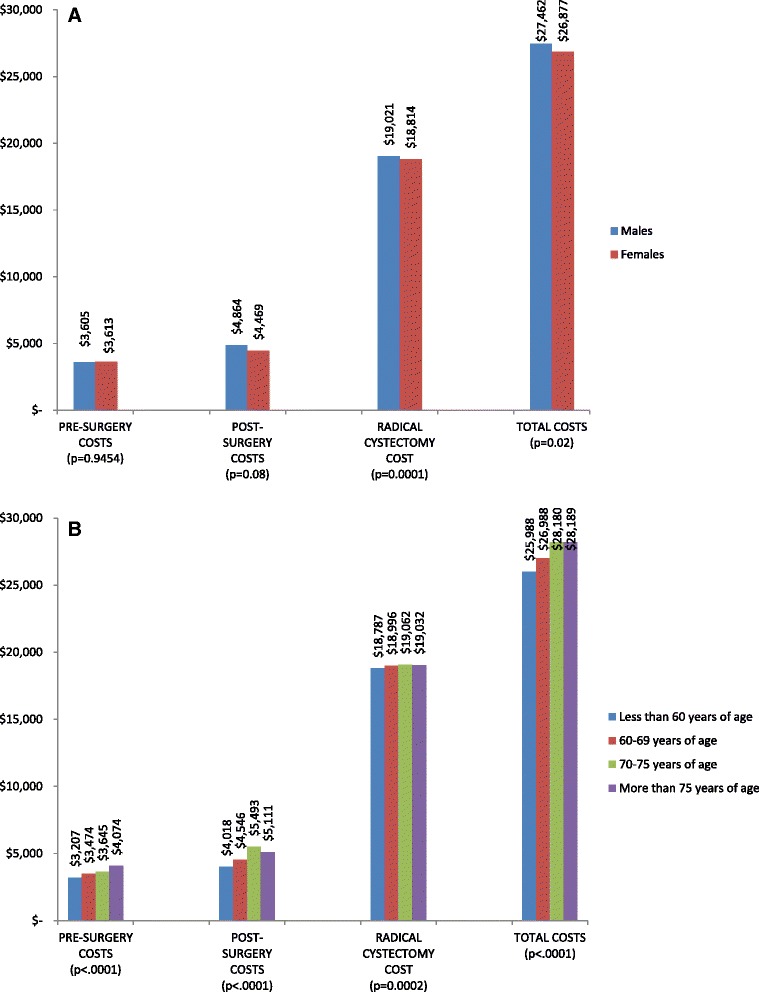
Average pre and post-surgery costs, radical cystectomy and total costs according to patient’s gender (**a**) and age (**b**)

Average costs per patient and mean units of medical services for each component are summarized in Table 3. Figure 1, Fig. 2(a) and (b) illustrate the weighted average of cost components for the pre and post-operative periods, and costs according to patient’s gender and age, respectively. Patients had a mean of 4.7 consultations with other specialists before their first urologist visit (average costs of pre-urologist consultations per patient: 1006 $). Mean number of urologist consultations and TURBTs in the four months before RC was 4.7 and 1.2 respectively. The average cost of TURBT was the most significant component of costs during the pre-operative period (2159 $), representing 40 % of total average costs before surgery. Patients tended to have more expensive post-operative urologist consultations compared to the pre-operative period (average cost of 437 $ per patient in the post-operative period compared to 162 $ in the pre-operative period). Imaging procedures were also used more frequently and at a higher cost after surgery (4.5 post-operative imaging procedures per patient with an average cost of 395 $). No significant differences were observed for these components of costs according to gender (Fig. 2(a)). On the other hand, older patients tended to have higher pre and post-operative costs (Fig. 2(b)).

**Table 3:** Average costs per patient attributable to the health continuum of care for bladder cancer. (Time window: from four months before radical cystectomy up to 1 year post surgery. Costs shown in Canadian dollars)

	N (%)	Mean and standard deviation (SD) of service units per patient	Median (IQR) service units per patient	Mean and standard deviation (SD) of costs per patient (Canadian dollars)	Median and range of costs per patient (Canadian dollars)
PRE-SURGERY COSTS (n = 2759)					
Pre-urologist consultations (all specialties confounded, excluding urologists)	2332 (84.5 %)	Mean: 4.7 (SD: 5.17)	Median: 3 (IQR: 2–6)	Mean: 1006 (1299)	Median: 450 (10–7834)
Post-urologist consultations (all specialties confounded, excluding urologists)	2155 (78.1 %)	Mean: 4.3 (SD: 4.2)	Median: 3 (IQR:2–5)	Mean: 935 (1038)	Median: 523 (6.2-6000)
Urologist consultations	2731 (99 %)	Mean: 4.7 (SD: 3.8)	Median: 4 (IQR:3–6)	Mean: 162 (80)	Median: 140 (17–1959)
Cystoscopies	1896 (68.7 %)	Mean: 1.19 (SD: 0.46)	Median: 1 (IQR: 1–1)	Mean: 323 (37)	Median: 273 (273–870)
TURBTs	2058 (74.6 %)	Mean: 1.15 (SD: 0.39)	Median: 1 (IQR:1–1)	Mean: 2159 (773)	Median: 1911 (1703–4115)
Imaging (physician fees)	2532 (91.7 %)	Mean: 2.5 (SD: 1.55)	Median: 2 (IQR: 1–3)	Mean: 242 (154)	Median: 205 (12-1397
RADICAL CYSTECTOMY COSTS					
Radical cystectomy	2759 (100 %)	1	1	Mean: 18 979 (1168)	Median: 18 440 (16 005–25 684)
POST-SURGERY COSTS					
Post-operative urologist consultations	2606 (94.5 %)	Mean: 4.7 (SD: 5.17)	Median: 3 (IQR: 2–6)	Mean: 437 (635)	Median: 196 (17–9479)
Post-operative consultations (all specialties confounded, excluding urologists and medical oncologists)	2742 (99.4 %)	Mean: 32 (SD: 29)	Median: 24 (IQR: 13–41)	Mean: 2232 (2275)	Median: 1464 (17–27057)
Post-operative medical oncologist consultations	809 (29.3 %)	Mean: 10 (SD: 10.9)	Median: 7 (IQR: 2–14)	Mean: 526 (579)	Median: 346 (14.75-3860)
Post-operative imaging (physician fees)	2535 (92.1 %)	Mean: 4.5 (SD: 3.3)	Median: 4 (IQR: 2–6)	Mean: 395 (307)	Median: 311 (12.3-2845)
Post-operative complications	811 (29.4 %)	Mean: 3.7 (SD: 3.36)	Median: 3 (IQR: 2–5)	Mean: 5703 (2076)	Median: 5062 (4017–18878)

### Cost of radical cystectomy

**Fig 3:**
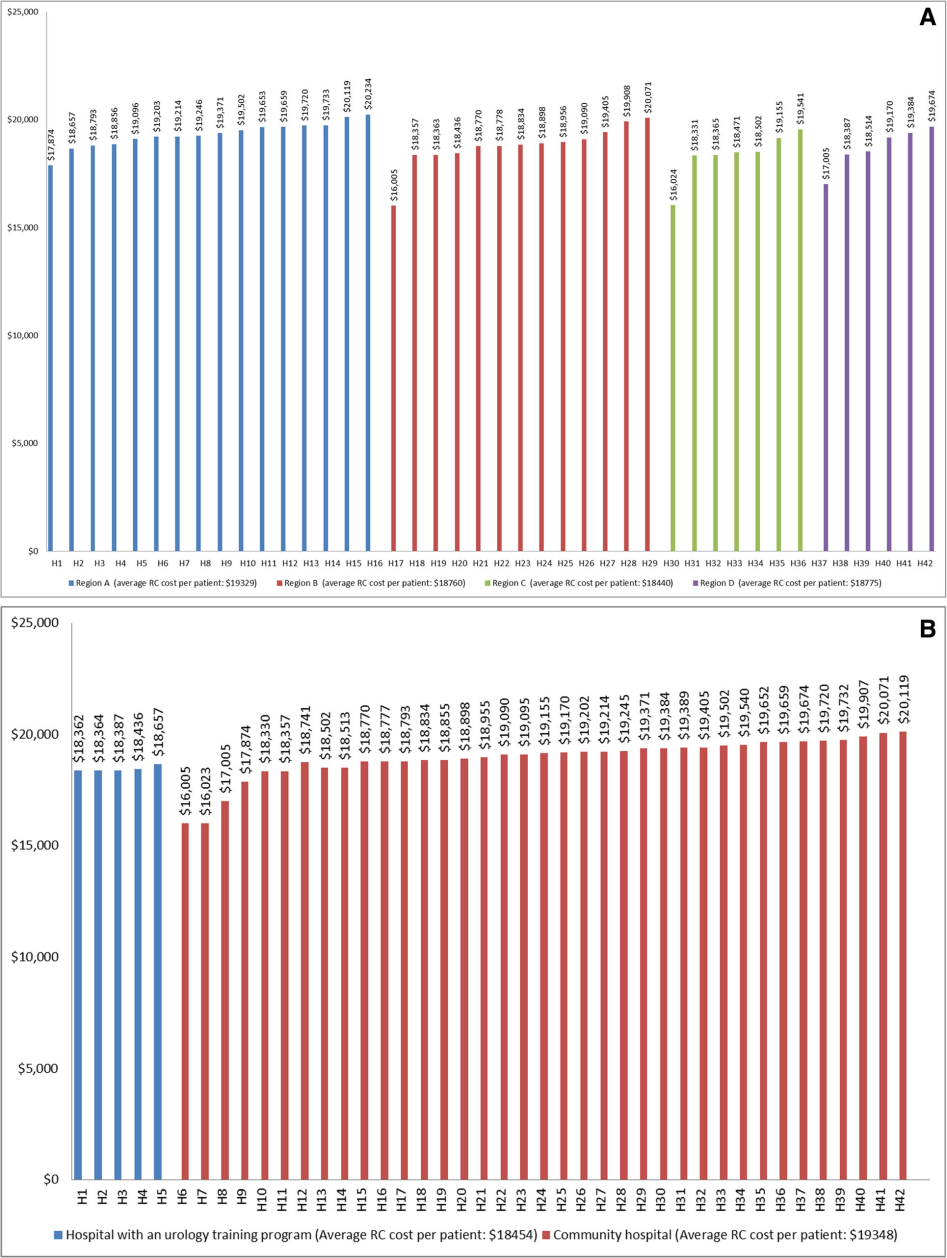
Variations for average RC costs among hospitals across the province (**a**), and according to the presence of a urology teaching program (**b**)

Cost of RC is shown in Table 3, and graphically illustrated in Figs. 3((a) and (b)). Average cost of surgery per patient was estimated at 18 979 $. Surgery was 207 $ more expensive in men compared to women (Fig. 2(a), p < .0001), and more costly in older patients (19 032 $ Fig. 2(b), p = 0.0002). Region A was the only administrative region with hospitals performing RC at a cost higher than the average (19 329 $, Fig. 3(a), p < .0001). Hospitals hosting a urology training program performed RC at an average of 1000 $ less expensive (at 5 %), compared to community hospitals (Fig. 3(b), p < .0001).

### Cost of post-operative complications

**Fig. 4:**
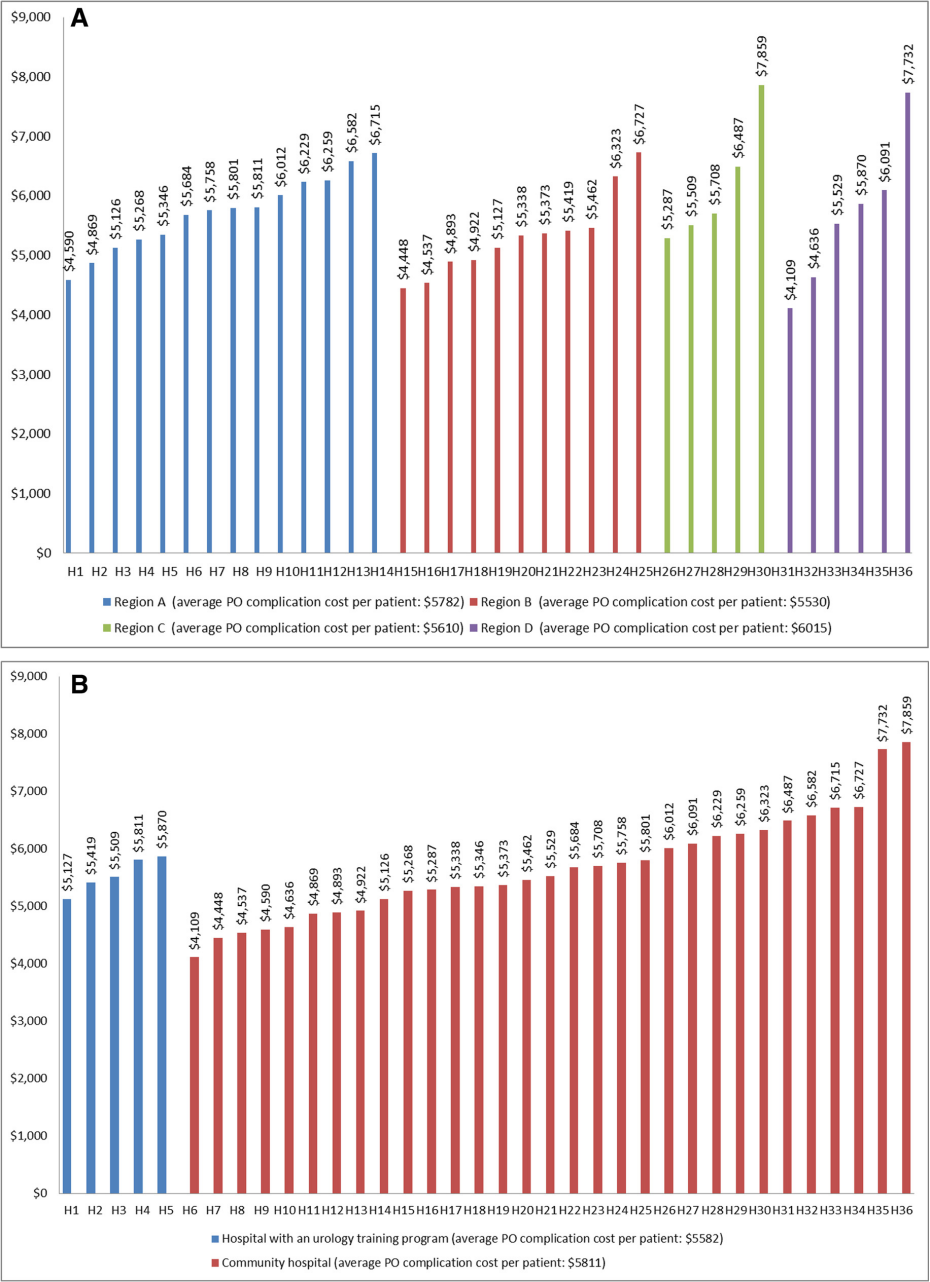
Variations for average post-operative complications costs among hospitals across the province (**a**), and according to the presence of a urology teaching program (**b**)

Cost of post-operative complications is shown in Table 3, and graphically illustrated in Fig. 1, Fig. (4(a) and (b)). Average cost of post-operative complications was the most significant component of costs after surgery. Among patients who had these complications (30 %); patients had a mean of 3.7 complications at an average cost of 5703 $ per patient. An extreme variation in the average costs per patient was identified among hospitals across the province, with hospitals of the Region D having the higher average costs per patient (6015 $, compared to 5782 $ in region A, 5530 $ in region B and 5610 $ in region C). Hospitals hosting a urology teaching program showed a non-significant lower average costs per patient for post-operative complications, compared to community hospitals (5582 $ and 5811 $, respectively).

### Total costs of bladder cancer

**Fig 5:**
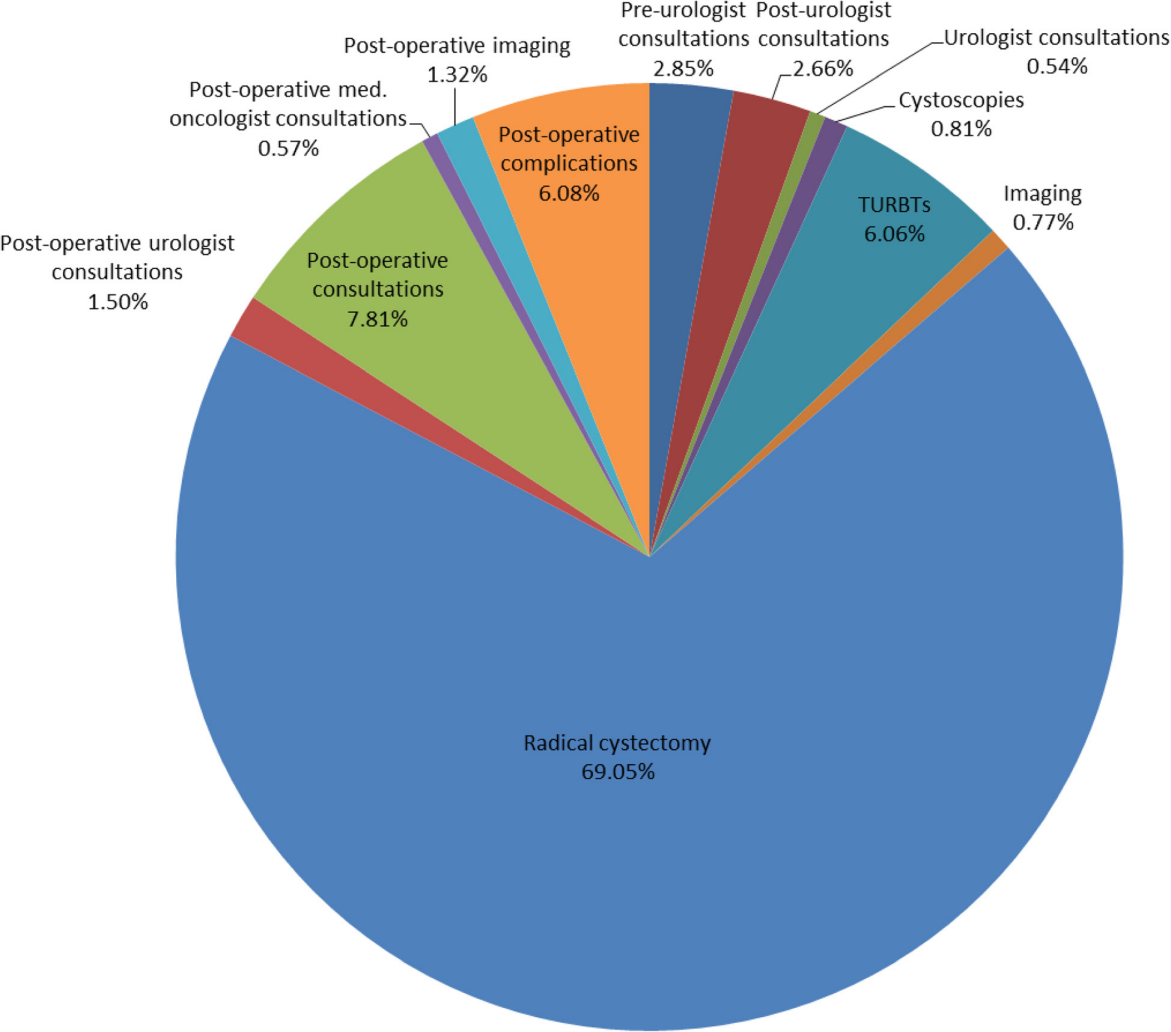
Percentage of each component of cost in the continuum of care for bladder cancer in Quebec

Units of health services utilization and its total costs are described in Table 4. Table 5 displays average and total RC costs per patient, average and total pre-surgery cost per patient and average and total post-surgery costs per patient stratified by year. Percentages of each component of costs are graphically represented in Fig. 5. Over 10 years, total costs of BC requiring radical cystectomy to the provincial health system was estimated at 75 563 893 $. During the study period, 2759 RCs were performed at a cost of 52 372 057 $, representing 69 % of total costs of RC for BC (Fig. 5). Post-operative consultations was the second most expensive component (5 594 774 $. 7 % of total costs) followed by post-operative complications (4 356 127 $, 6 % of BC total costs) and TURBTs (4 340 247 $, 6 % of total costs).

**Table 4:** Health-care services utilization and total costs associated with bladder cancer. (Time window: from four months before radical cystectomy up to 1 year post surgery; Costs in Canadian dollars)

	N (% of the total study population)	Total Units	Total costs (Canadian dollars)
PRE-SURGERY COSTS (n = 2759)			
Pre-urologist consultations (all specialties confounded)	2332 (84.5 %)	9931	2 040 407
Post-urologist consultations (all specialties confounded)	2155 (78.1 %)	8853	1 906 147
Urologist consultations	2731 (99 %)	10 975	385 244
Cystoscopies	1896 (68.7 %)	2103	577 135
TURBTs	2058 (74.6 %)	2274	4 340 247
Imaging (physician fees)	2532 (91.7 %)	5658	551 684
RADICAL CYSTECTOMY COSTS			
Radical cystectomy	2759 (100 %)	2759	52 372 057
POST-SURGERY COSTS			
Post-operative urologist consultations	2606 (94.5 %)	22 697	1 073 025
Post-operative consultations (all specialties confounded)	2742 (99.4 %)	81 562	5 594 774
Post-operative medical oncologist consultations	809 (29.3 %)	7728	406 170
Post-operative imaging (physician fees)	2535 (92.1 %)	10 716	945 316
Post-operative complications	811 (29.4 %)	2839	4 356 127

**Table 5:** Costs associated with bladder cancer stratified by year of surgical procedure. (Time window: from four months before radical cystectomy up to 1 year post surgery; Costs in Canadian dollars)

Year	Number of RC performed by year	Average RC cost per patient by year	Total RC cost per year	Average pre-surgery costs per patient by year	Total pre-surgery costs per year	Average post-surgery costs per patient by year	Total post-surgery costs by year	Average total BC costs per patient by year	Total BC costs by year
2000	268	19 048	5 104 977	3436	921 024	3908	1 047 425	26 393	7 073 427
2001	281	19 246	5 408 320	3516	988 186	4248	1 193 806	27 011	7 590 313
2002	265	19 284	5 110 398	3296	870 384	4475	1 176 988	27 010	7 157 771
2003	260	19 214	4 995 825	3334	867 014	4752	1 230 848	27 283	7 093 688
2004	289	19 207	5 551 009	3725	1 076 586	5243	1 504 842	28 139	8 132 438
2005	297	19 133	5 682 666	3743	1 108 099	5458	1 621 090	28 322	8 411 856
2006	282	18 833	5 311 004	3708	1 045 657	5938	1 674 592	28 479	8 031 253
2007	282	18 582	5 240 391	3693	1 041 576	5600	1 573 614	27 856	7 855 582
2008	278	18 645	5 183 322	3609	1 003 438	5302	1 463 482	27 518	7 650 243
2009	257	18 615	4 784 141	3717	955 341	3298	827 833	25 553	6 567 317
Total	2759	18 979	52 372 057	3762	9 877 310	4770	13 314 525	27 388	75 563 893

### Time series and trend analyses

**Fig 6:**
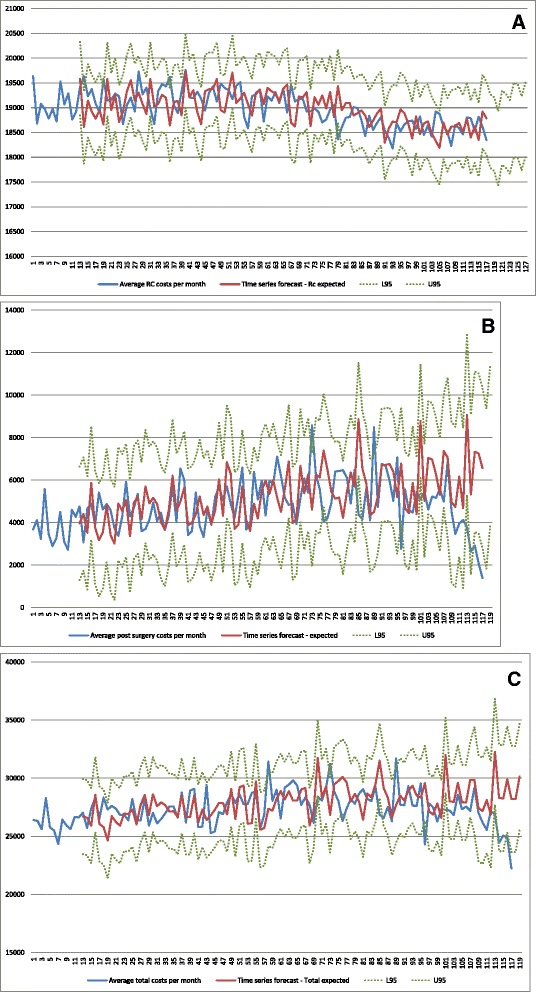
Time series trends in cost per patient (y axis) over time (x axis): (**a**) Average RC costs; (**b**) Average post-operative costs; (**c**) Average total costs *

Figure 6 demonstrates time trends in average RC costs per patient (A), average post-operative costs per patient (B) and average total costs per patient (C) over the 117 month period between January 2000 and September 2009. Trends in average RC costs per month were described by an ARIMA (2,1,1) model, with comparison between observed and forecasted values modeled from period 72 (January 2006), and forecasted up to period 117. Period 72 was chosen as a cut-off point because of an apparent decreasing trend for this component of cost starting from this period, as inspected at the time series plot for this component of cost. No significant trend was detected over time (Fig. 6(a)). On the other hand, we observed a non-significant decreased trend during the year 2009 for the average of post-operative and total costs per month, as described by two independent ARIMA (1,1,0) models (Fig. 6(b) and (c)). A significant difference of −5175 $ was detected between observed and predicted values of average post-operative costs in September 2009. In addition, a significant difference of −6000 $ was detected between observed and predicted average total costs in September 2009. These decreases in costs are attributed to a lower average cost of medical consultations, compared to the mean for the entire study period (Table 6). Stationarity analyses did not reveal any time trend for the number of RC performed during each month period, as well as for average pre-operative costs per month.

**Table 6:** Average costs for consultations in September 2009, compared to average forecasted values from ARIMA (1,1,0) model, and average costs for the study period

	Mean and standard deviation (SD) of costs for September 2009 (Canadian dollars)	Median and range of costs for September 2009 (Canadian dollars)	Mean and standard deviation (SD) of costs over all study period (Canadian dollars)	Median and range of costs over all study period (Canadian dollars)	Predicted costs values for September 2009 – ARIMA (1,1,0)
Pre-urologist consultations (all specialties confounded)	Mean: 667 (825)	Median: 362 (20–3156)	Mean: 1006 (1299)	Median: 450 (10–7834)	*
Post-urologist consultations (all specialties confounded)	Mean: 712 (569)	Median: 428 (104–3511)	Mean: 935 (1038)	Median: 523 (6.2-6000)	*
Urologist consultations	Mean: 127 (85)	Median: 129 (16.9-245)	Mean: 162 (80)	Median: 140 (17–1959)	*
Post-operative consultations (all specialties confounded)	Mean: 453 (345)	Median: 261 (64–1724)	Mean: 2232 (2275)	Median: 1464 (17–27057)	*
Post-operative imaging	Mean: 139 (326)	Median: 90 (31.6-496)	Mean: 395 (307)	Median: 311 (12.3-2845)	*
Post-operative costs	Mean: 1384 (589)	Median: 278 (57.8-11615)	Mean: 4770 (5220)	Median: 2759 (33.8-42000)	6559 (95 % CI: 1827–9366)
Total costs	Mean: 22223 (19560)	Median: 21075 (17341–33249)	Mean: 27326 (18652)	Median: 25928 (17222–62000)	28224 (95 % CI: 23654–32794)

* No calculation performed.

## Discussion

Previous studies have shown that radical cystectomy accounts for the largest proportion of payments for bladder cancer care; these costs showing great variability depending on the country [8, 16]. For example, average European RC reimbursement costs in US dollars, (including hospitalizations costs and medical fees) varies from 5684 $ in the United Kingdom, 9697 $ in France, 10 932 $ in Belgium and 15 419 $ in Germany [7]. The cost of radical cystectomy including lymphadenectomy and urinary diversion at an academic hospital center in the USA was calculated at 30 000 $ with most costs attributed to the operating room and hospital stay [8]. In our study, RC costs in Quebec lies between the estimates for Europe and North America and represents 69 % of average costs of bladder cancer requiring RC from diagnosis to one year after surgery. This variation in surgery costs estimates across countries is probably attributable to differences in practices such as inpatient or outpatient care, duration of hospitalization, methods of calculating costs and billing, disease incidence, and the type of surgical procedure (open, laparoscopic, or robot-assisted) [17].

Our findings also showed variations in costs for RC within the provincial health-care system. Costs of surgery varied across patient gender, patient age group, and administrative region where RC was performed. Cost variations were also detected across individual hospitals, with the majority of facilities performing surgeries at a higher than the average cost. Although these disparities were relatively small, we detected a significant 1000 $ greater average cost per patient between community hospitals compared to hospitals hosting a urology training program. Given that hospitalization and operation room costs were estimated with the provincial ministry of health’s data and attributed equally to all surgical procedures in all hospitals in our cohort, we believe that this difference is caused by the greater amount of medical fees reimbursed to physicians in community hospitals. Indeed, we found that among community hospitals, surgeries are performed by an average of 2.9 surgeons (SD: 1.15), compared to 1.76 (SD: 0.9) among hospitals hosting a urology training program. This is likely explained by the fact that in academic centers the primary surgeon is often assisted by resident house staff that does not require reimbursement by the RAMQ, as opposed to community hospitals where 2 or more surgeons are involved, each billing the RAMQ. It has been reported that logistical difficulties inherent to the additional burden of excess travel time for patients and physicians and the potential marginalization of lower-volume hospitals may increase the indirect costs attributable to surgery in community hospitals [18]. Our results may be useful in identifying potential geographic disparities in the cost of BC cancer care.

Given that BC requires patients to undergo laboratory tests and invasive procedures for diagnosis, much of the costs during the management of the disease are attributed to cystoscopy and TURBT [19]. The frequent number of these procedures among bladder cancer patients results in significant cumulative costs [20]. Reimbursement for cystoscopy in the USA by Medicare is approximately 223 $ [8]. Average costs of cystoscopy in our study (323 $) were higher than some European countries with similar publically funded health system, such as Italy (76 $), Germany (61 $), France (51 $), but considerably lower than UK (620 $) [7]. On the other hand, average costs of TURBT in Quebec (2159 $) showed to be similar to these countries (UK: 2154 $; Germany: 1967 $, France: 1124 $, Italy: 2741 $ and Belgium: 2201 $) [7]. TURBT represents the first line of treatment for new tumors and accounts for a substantial portion of total bladder treatment costs [6]. In the USA, after accounting for costs of anesthesia and the operating room, these costs were estimated to be higher than 2900 $ [21]. Costs at an academic US medical center ranged from 3000 $ to 6000 $ depending on patients average hospitalization duration [8]. In this study, the mean length of stay was 2 days which contributed to this variation of TURBT costs. In some centers, patients spend an average of 4 days in the hospital after a TURBT. One day surgery is common in Quebec [13, 14].

Surgery for bladder cancer carries a high risk for perioperative and post-operative complications with rates varying from 20 % to 60 % [22, 23]. A recent study conducted by our group estimated that in Quebec postoperative complications occur in 30 % of RC cases [12]. Complications of radical cystectomy prolong the patient’s length of hospital stay and significantly increase the total bladder cancer costs [24]. Estimated costs of complications vary greatly across different studies according to definitions and categorization of post-operative complications, and consequently duration of hospital stay. One of the highest costs attributable to an adverse event after cystectomy was shown for sepsis in the USA [25]. In this study, length of stay for patients with septicemia was estimated at 29 days compared with 10 days for controls. Hospital charges for bacterial infections was 107 724 $. Average costs for selected post-operative complications [12] in our study was estimated at 5703 $, which is lower than other average estimates in the literature [8]. A great variation in costs was observed between hospitals in Quebec with a range of 4109 $ - 7732 $. Similar to what was detected for RC average costs, community hospitals showed higher average post-operative complication costs per patients, compared to academic hospitals. More studies investigating predictors of higher costs and the impact of cost disparities and its relation to outcome and survival are needed.

Average total costs per patient in Quebec was found to be lower than the Canadian province of Ontario (27 388 $ in Quebec versus 33 759 $ in Ontario) [26]. Although we cannot establish causal relations between medical costs and patient outcomes based on such ecological data, it is noteworthy that 5-year overall survival after RC in Quebec is 46 %, while the rate is 35 % in Ontario [18].

During the study period, total costs of bladder cancer requiring RC from diagnosis up to one year of follow up were estimated at more than 70 million dollars, with more than 70 % of this total expenditure attributed to health care associated to surgery. Total costs of surgery and post-operative complications were estimated at 50 million dollars and 4 million dollars, respectively. Total costs in our cohort represent a small fraction of what was estimated in the USA for 2010 (3.98 billion dollars, for all cases confounded) [27]. Reasons for the rising cost of bladder cancer in the USA are attributable to the fact that most patients in the US are part of the Medicare Program [1]. In addition, Medicare reviewed its reimbursement fees for office-based endoscopic procedures in 2005, which led to an increased number of bladder lesions detected and a higher total number of bladder cancer-related procedures being performed [28]. Total costs estimates in the United Kingdom included estimates of indirect costs from loss of earnings and reached 125.2 million in 2010 [29]. Cost of the annual medical care was estimated to be about 1 million dollars in Sweden [19] and 27 million dollars in South Korea [30]. These remarkable variations are due to differences in both disease incidence and costs of per patient treatment in each national health system. Analyses of specific explanatory differences in clinical structure or the availability of resources have not been extensively investigated [1]. A noteworthy result of our study was a decreasing trend in the average post-operative costs and total costs caused by a lower average cost for consultations observed in the year 2009.

To our knowledge, this is the first study to describe costs attributed to the number of pre and post-urologist consultations to general practitioners and other specialists, as well as the costs associated to urologist consultation during the pre and post-operative period. Given the paucity of detailed descriptive studies on the health economics of BC in Canada [26], our results provide some evidence-based data of interest to health care providers and policy-makers to better understand the relationship between resource-utilization and costs associated with the disease, and to improve the efficiency and outcomes.

This study had some significant limitations inherent to the use of administrative databases. We were unable to measure some factors that can play an important role on costs of BC requiring RC, such as grade, stage and severity of the tumor. Data on comorbidity and patient functional status are also lacking. More importantly, since we did not know the actual hospitalization period for each patient undergoing RC, we could not be more precise in attributing costs to RC and post-operative complications. The actual variance of health services costs is hence, mainly due to the overall differences in billing codes for physicians’ payments. This information would be very important in order to compare costs and efficiencies between administrative regions, individual hospitals, and surgeons. The imputation of some economic component for medical services may have caused some degree of information bias in the calculation of average costs per patient. Nevertheless, if information bias is present, it is certainly non-differential, which not undermine internal validity of our findings. Given that we do not have data on some medical services such as prescriptions filled by patients, we could not compare our findings with an external cohort of patients that would serve as control group for estimation and comparison of attributable and net costs. Moreover, considering the intrinsic discrepancies in the management of BC across different health care systems, we are not convinced that comparing of our findings in the province of Quebec with a different “control cohort” would decrease the possibility of information bias.

Also, our study did not account for the burden of indirect costs in our analyses, for which we acknowledge that these costs in the form of patients’ and caregivers’ time, as well as reduced physical and social functioning contribute to the overall burden of bladder cancer on society. On the other hand, the fact that the RAMQ is single-payer and public funded system with universal healthcare coverage allows the collection of prospective information for a large sample size, which increases external validity of our findings. The linkage between the two databases was done using a unique patient identifier, which permitted a very reliable correspondence of medical services data [31].

## Conclusion

Costs of RC surgery, TURBT, medical consultations, and post-operative complications were the most important economic components of total bladder cancer cost per patient requiring radical cystectomy in Quebec. Significant variations in costs were detected between academic and community hospital, as well as between geo-administrative regions across the province. More studies are needed to evaluate predictors of costs and the impact of bladder cancer expenditures on patient’s clinic outcome and survival.

## Abbreviations

BC: bladder cancer
RC: radical cystectomy
TURBT: transurethral resection of the bladder tumor
RAMQ: *Régie de l’assurance maladie du Québec*
ISQ: *Institut de la statistique du Québec*
CAI: *Comission de l’acces a l’information*
MSSS: *Ministere de la Sante et des Services Sociaux*
ICD-9: International classification of diseases – 9th edition
ARIMA: autoregressive integrated moving average
